# Physiological constraints to climate warming in fish follow principles of plastic floors and concrete ceilings

**DOI:** 10.1038/ncomms11447

**Published:** 2016-05-17

**Authors:** Erik Sandblom, Timothy D. Clark, Albin Gräns, Andreas Ekström, Jeroen Brijs, L. Fredrik Sundström, Anne Odelström, Anders Adill, Teija Aho, Fredrik Jutfelt

**Affiliations:** 1 Department of Biological and Environmental Sciences, University of Gothenburg, Box 463, Gothenburg 405 30, Sweden; 2 University of Tasmania and CSIRO Agriculture Flagship, Tasmania, Hobart 7000, Australia; 3 Department of Animal Environment and Health, Swedish University of Agricultural Sciences, Box 234, Skara 532 23, Sweden; 4 Department of Animal Ecology/Evolutionary Biology Centre, Uppsala University, Box 592, Uppsala 751 24, Sweden; 5 Department of Aquatic Resources, Institute of Coastal Research, Swedish University of Agricultural Sciences, Skolgatan 6, Öregrund 742 42, Sweden; 6 Department of Biology, Norwegian University of Science and Technology, Trondheim NO-7491, Norway

## Abstract

Understanding the resilience of aquatic ectothermic animals to climate warming has been hindered by the absence of experimental systems experiencing warming across relevant timescales (for example, decades). Here, we examine European perch (*Perca fluviatilis, L.*) from the Biotest enclosure, a unique coastal ecosystem that maintains natural thermal fluctuations but has been warmed by 5–10 °C by a nuclear power plant for over three decades. We show that Biotest perch grow faster and display thermally compensated resting cardiorespiratory functions compared with reference perch living at natural temperatures in adjacent waters. However, maximum cardiorespiratory capacities and heat tolerance limits exhibit limited or no thermal compensation when compared with acutely heated reference perch. We propose that while basal energy requirements and resting cardiorespiratory functions (floors) are thermally plastic, maximum capacities and upper critical heat limits (ceilings) are much less flexible and thus will limit the adaptive capacity of fishes in a warming climate.

## Abstract (web summary)

Understanding climatic adaptation in fish is limited by a lack of large-scale, long term acclimation studies. Here, Sandblom *et al*. show that fish exposed to a 5-10 °C increase in water temperature next to a nuclear power plant display contrasting upper and lower cardiorespiratory thermal compensation limits.

## Graphical abstract

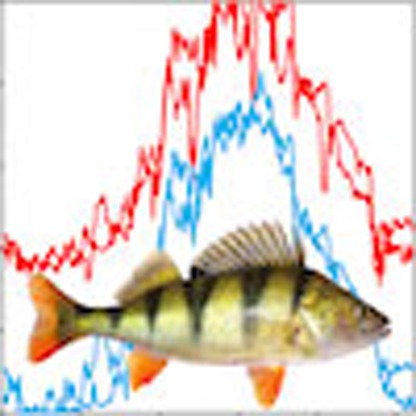

Aquatic ectotherms may suffer significant population declines or shifts in latitudinal distribution as climate warming challenges their limits of thermal tolerance^1^,^2^. However, inter-species differences in sensitivity to climate change are considerable, as some species are more eurythermal and possess a greater capacity to produce phenotypes with the physiological and biochemical traits necessary to tolerate a warmer environment. Although such adjustments can either be accomplished through individual phenotypic plasticity (that is, acclimation or acclimatization)^3^ or genetic adaptation across generations^4^,^5^,^6^, the underlying physiological attributes determining species biogeography and thermal tolerance remain insufficiently explored^7^. Moreover, limited knowledge exists on the differential thermal plasticity in physiological traits across species and individuals, yet this is crucial for identifying thermal constraints and ‘physiological bottlenecks' to climate change resilience. These knowledge gaps are partly due to a lack of model systems where warming has prevailed over relevant timescales (that is, decades and beyond) and with natural temperature fluctuations, where limits to physiological acclimation and adaptation can be examined following generations of chronic warming^8^,^9^.

Two prominent hypotheses exist in the current literature regarding the general physiological underpinnings associated with species biogeography and thermal tolerance of ectothermic animals. The first hypothesis suggests that warming causes a gradual decline in physiological performance, which is explained by the maximum oxygen consumption rate (_2max_) under steady-state conditions failing to keep pace with the rate of increase in resting oxygen consumption rate (Ṁ_2rest_) as the environment warms, consequently resulting in a progressive decline in aerobic scope (AS; that is, 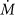
_2max_–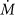
_2rest_)^10^. This hypothesis further assumes that fitness and performance traits (for example, growth, reproduction, aerobic swimming capacity) gradually decline beyond optimal temperatures as a consequence of the proposed decline in AS. The second hypothesis is based on macrophysiological observations across phyla and suggests that relatively rare extreme events such as episodic heat waves exceeding an animal's critical thermal maximum (CT_max_), rather than higher average environmental temperature, may be more important in shaping population performance and biogeography with respect to environmental warming^11^,^12^. Although some studies suggest that thermal constraints may arise at the organism and ecosystem level before CT_max_ is reached^13^, a reduced difference between a species' habitat temperature (*T*_hab_) and CT_max_ with global warming is thought to represent a reduced warming tolerance (warming tolerance*=*CT_max_*−T*_hab_), consequently decreasing the buffer capacity against transient warm episodes^14^,^15^.

Despite the recent popularity of each of these hypotheses, the ideas remain speculative as relatively little is known about the long-term thermal plasticity of the physiological traits on which these hypotheses are based (that is, cardiorespiratory scopes and CT_max_). Although the CT_max_ of marine fishes is typically closely correlated with their current latitudinal distribution and habitat temperature^13^, the limits to plasticity in thermal tolerance with future warming of the seas are virtually unknown. Indeed, upper thermal tolerance limits are suggested to be evolutionarily and plastically rigid in terrestrial ectotherms^16^,^17^, and short-term warm acclimation in fishes may only partially compensate CT_max_ such that warming tolerance is reduced at higher acclimation temperatures^18^. Moreover, recent short-term laboratory acclimation experiments on fishes have revealed interesting metabolic patterns where the thermal plasticity of AS can be substantial because of pronounced changes in 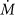
_2rest_, whereas 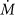
_2max_ remains largely unchanged or even decreases with warm acclimation^19^,^20^. However, it is presently unknown how these respiratory functions are affected by chronic warming and what the underlying mechanisms are.

Here, we utilized a unique, artificially heated ecosystem in the Baltic Sea to enhance our understanding of the aforementioned ideas. The Biotest enclosure (Fig. 1a,b) has received heated water from a nearby nuclear power plant for over three decades, maintaining temperatures ∼5–10 °C above the surrounding sea but retaining daily and seasonal variations in temperature (Fig. 1c). Using an abundant eurythermal temperate fish species as our model (European perch, *Perca fluviatilis, L.*), we hypothesized that physiological *‘floors'* such as resting cardiorespiratory functions would exhibit considerable thermal plasticity in the chronically heated Biotest perch, whereas the physiological *‘ceilings'* such as maximum cardiorespiratory capacities and CT_max_ would display little thermal plasticity. We refer to this hypothesis as ‘plastic floors and concrete ceilings'.

We show that Biotest fish grow faster at early ages and exhibit morphological changes as adults including reduced relative organ masses. Consistent with our hypothesis, basal energy requirements (that is, _2rest_) and resting cardiovascular functions show considerable thermal compensation and are markedly reduced in the chronically heated Biotest perch when compared with acutely heated reference perch from cooler adjacent waters. Maximum cardiorespiratory traits and CT_max_ exhibit limited or no thermal compensation in Biotest perch, leading to a markedly reduced warming tolerance and presumably a greater vulnerability to transient heat waves. This study lends further support to the hypothesis of ‘plastic floors and concrete ceilings', and shows that the true sensitivity of a population to warming may be underestimated when only resting physiological functions are considered.

## Results and Discussion

### Effects of chronic warming on fish growth and morphology

**Figure 1:**
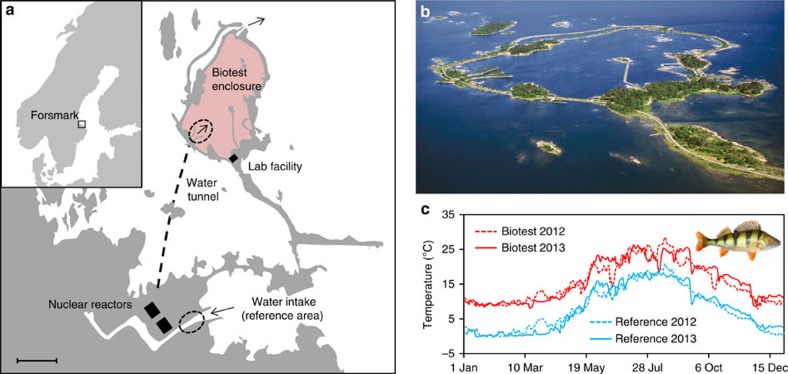
Biotest area overview and thermal conditions. Schematic map (**a**) and aerial photograph (**b**) of the Biotest enclosure (Photo: Göran Hansson, used with permission). Depicted in **a** are the cooling water intake channel and tunnel under the seabed (hatched line) supplying heated cooling water into the Biotest enclosure. Hatched ellipses show areas where experimental fish were collected and the scale bar represents 500 m. The inset in **a** displays Scandinavia and the location of the experimental facility in Forsmark along the Swedish Baltic Sea coast (for further details see online supporting material). Panel **c** shows yearly temperature profiles with daily mean temperatures for the reference area (blue) and the Biotest enclosure (red) in 2012 (hatched lines) and 2013 (solid lines). Inset in **c** shows the experimental species European perch (*Perca fluviatilis, L.*; Photo: Fredrik Jutfelt, used with permission).

We compared yearly growth for chronically warmed fish from the Biotest enclosure with reference fish living in adjacent waters under natural thermal conditions (see Fig. 1a and the Methods for details). Chronically warmed fish were significantly longer than reference fish at 2–4 years of age, which was explained by a faster growth rate during the second and third year of life. In older age classes, this difference in growth rate disappeared (Supplementary Fig. 1), possibly due to the earlier shift in energy investment from growth to reproduction observed in Biotest fish^21^. Although there was no difference in body condition between the two populations in late summer when the fish were collected, the chronically warmed fish had significantly smaller relative organ masses (Supplementary Table 2). Collectively, with recent studies revealing genetic differentiation^22^, these findings provide compelling evidence that the two populations of perch have been functionally separated for at least their lifetime and probably for several generations. An interesting avenue for future studies will be to decipher the role of elevated temperature *per se* on ecological feedbacks (for example, food availability and composition) in the context of the observed morphological and physiological differences. Even so, the perch population in the Biotest enclosure represents an unprecedented model to examine the long-term physiological responses of temperate fishes facing a severe climate warming scenario under ecologically realistic conditions (that is, RCP 8.5 forecasts 5–7 °C warming of the coastal Baltic Sea by 2100 (ref. 23)).

### Resting and maximal cardiorespiratory functions

**Figure 2:**
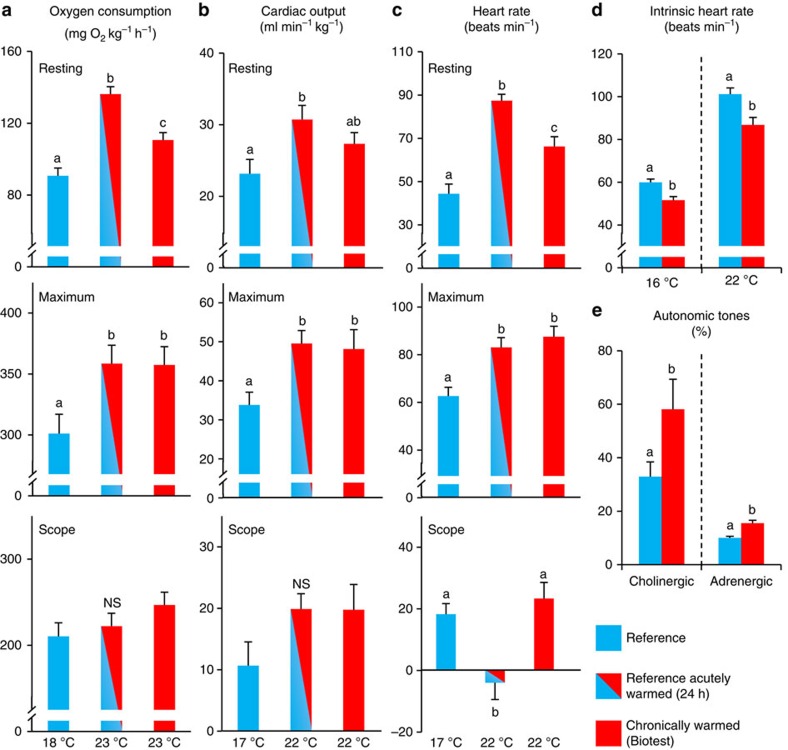
Cardiorespiratory responses to acute and chronic heating. Variables are oxygen consumption rate (**a**), cardiac output (**b**), heart rate (**c**), intrinsic heart rate after complete autonomic blockade (**d**) and cardiac cholinergic and adrenergic tones (**e**). Variables in **a**–**c** are presented for resting conditions and the maximum response after exhaustive exercise with scope being the difference between maximum and resting. Reference fish were tested at natural conditions (16–18 °C, blue, *n*=7–9) and 24 h after transfer to Biotest conditions (22–23 °C, red–blue, *n*=7–10). Biotest fish were tested at Biotest conditions (22–23 °C, red, *n*=8–10). Intrinsic heart rate was first tested at the respective environmental temperatures and then after acute transition to the opposite environmental temperatures (*n*=9–13). Autonomic tones were determined at the respective environmental temperatures (*n*=9–13). Values are means (+s.e.). Dissimilar letters represent significant differences among groups (*P*≤0.05; analysis of covariance with body mass as covariate in **a**, one-way analysis of variance in **b**,**c** and two-tailed Student's *t*-test in **d**,**e**).

Resting and maximum cardiorespiratory functions in reference perch were measured at natural temperatures and after 24 h of acute warming to the temperature of the Biotest enclosure. We then examined the long-term thermal plasticity of these traits by comparing the acutely warmed reference fish with Biotest fish following lifetime (and probably generational) acclimation to warming (see Supplementary Table 3 for details on experimental animals and protocols). In accordance with our hypothesis, 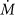
_2rest_ of reference fish increased by 48% when acutely warmed from 18 to 23 °C (*Q*_10_=2.2), yet the chronically warmed Biotest fish at 23 °C displayed a significant metabolic thermal compensation with a lower 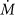
_2rest_ than acutely warmed reference fish at the same temperature (*Q*_10_=1.5; Fig. 2a and Supplementary Table 4). Further supporting our hypothesis, the significantly elevated 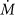
_2max_ of reference fish at 23 °C did not differ from chronically warmed Biotest fish at the same temperature, revealing little long-term thermal plasticity in 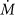
_2max_ (Fig. 2a).

Cardiovascular oxygen transport capacity is mechanistically linked with overall aerobic capacity^24^, and the maximum heart rate during rapid thermal ramping of anaesthetized Atlantic salmon (*Salmo salar*) subjected to lifetime warm acclimation is reported to be highly thermally plastic^25^. Therefore, we examined whether our hypothesis was also true for cardiovascular function. When reference fish were acutely warmed from 17 to 22 °C, resting cardiac output increased by 34% primarily through an increased heart rate (Fig. 2b,c), although there was a compensatory reduction in cardiac stroke volume after warming (Supplementary Table 4). Consistent with our hypothesis, resting cardiac output in chronically warmed Biotest fish exhibited considerable thermal compensation with a reduced *Q*_*1*0_ (1.3 versus 2.0) and it did not differ significantly from reference fish at natural temperatures. Moreover, resting heart rate was significantly lower in Biotest fish than in acutely warmed reference fish (Fig. 2b,c and Supplementary Table 4).

Again, maximum cardiac output and heart rate increased with temperature from 17 to 22 °C, but consistent with the response in 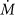
_2max_ there was no difference in maximum cardiovascular capacity between the acutely warmed reference fish and the chronically warmed Biotest fish. Similar patterns were evident for cardiac power output (Supplementary Table 4), revealing little thermal plasticity of maximum cardiovascular functions. Interestingly, both reference fish and chronically warmed fish altered their cardiac output through tachycardia in response to exercise at their respective environmental temperatures and had similar heart rate scopes. However, the mechanism for increasing cardiac output in acutely warmed reference fish differed markedly, as heart rate scope approached zero because of the dramatic increase in resting heart rate (Fig. 2c). Instead, the acutely warmed perch relied primarily on elevated stroke volume to increase blood flow (Supplementary Table 4).

### Neural and cellular mechanisms of cardiac thermal plasticity

The ability to switch from frequency- to volume-mediated control of cardiac output during an acute warming event, while maintaining maximum cardiac output and cardiac power output, indicates a highly thermally flexible cardiovascular phenotype in perch. Yet, the thermally induced adjustment of resting heart rate and the recovery of heart rate scope in chronically warmed fish reveals a clear and likely adaptive cardiovascular adjustment to the chronically warm conditions of the Biotest enclosure. In light of this interesting finding, we sought a mechanistic explanation for the reduced resting heart rate associated with the reduction in 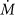
_2rest_ in chronically warmed fish.

*In vivo* heart rate is set by the balance between stimulating β-adrenergic and inhibitory cholinergic neurohumoral tone on the heart, as well as the spontaneous depolarization rate of the cardiac pacemaker (that is, the intrinsic heart rate)^26^. To examine the thermal plasticity of these controlling factors, we used pharmacological tools in cannulated individuals from both populations (see Supplementary Fig. 2 and Supplementary Table 3 for details). The inhibitory cholinergic tone was nearly doubled in chronically warmed Biotest fish, and the intrinsic pacemaker rate was significantly reduced across test temperatures (Fig. 2d,e). Thus, the thermal plasticity of resting heart rate is explained by a combination of increased neural inhibition of heart rate, as well as a reduction in the intrinsic pacemaker rate of chronically warm fish.

### Thermal plasticity of acute warming tolerance

**Figure 3:**
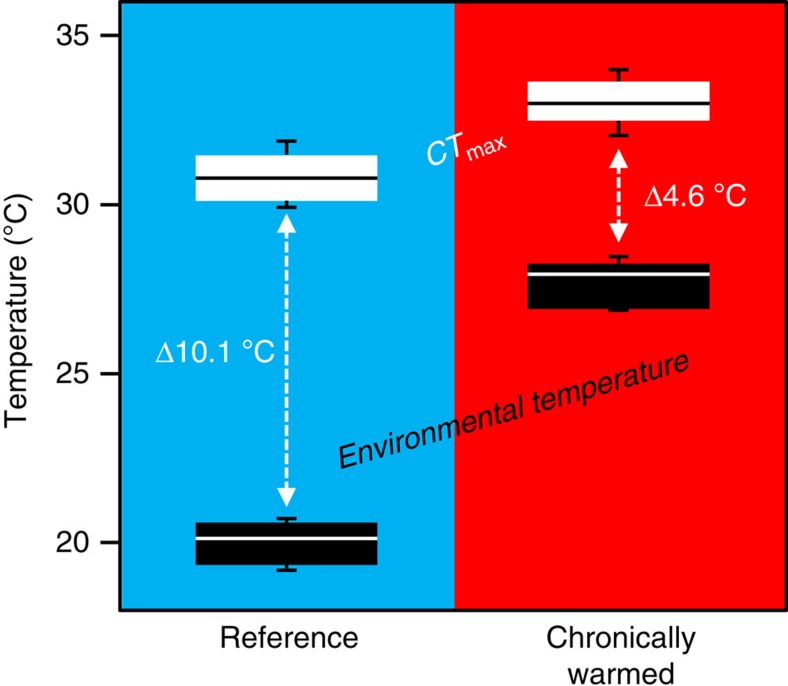
Warming tolerance limits. Thermal limits during summer peak temperatures under reference (blue, *n*=16) and chronically warmed conditions in the Biotest enclosure (red, *n*=16). The white boxplots show the upper critical temperature (CT_max_), with the whiskers representing the range, the box is the middle 50% of the data and the horizontal line is the median. Black boxplots represent the water temperature. The delta (Δ) values are the warming tolerance ranges.

Given the predictions that relatively fixed lethal temperature limits are key factors in shaping population performance and biogeography^11^,^12^, we examined whether the chronic warming of Biotest fish had affected acute heat tolerance limits. As expected, CT_max_ in chronically warmed fish was significantly higher (2.2 °C) than reference fish. However, the increase in CT_max_ was much smaller than the difference in environmental temperatures, leaving the Biotest fish with significantly reduced warming tolerance compared with reference fish (Fig. 3). These findings represent another facet of the hypothesis of ‘plastic floors and concrete ceilings', where variations in the basal or resting variables at different environmental temperatures are not reflected in similar compensations of the ‘ceilings' such as upper thermal tolerance limits.

### Synthesis and conclusions

**Figure 4:**
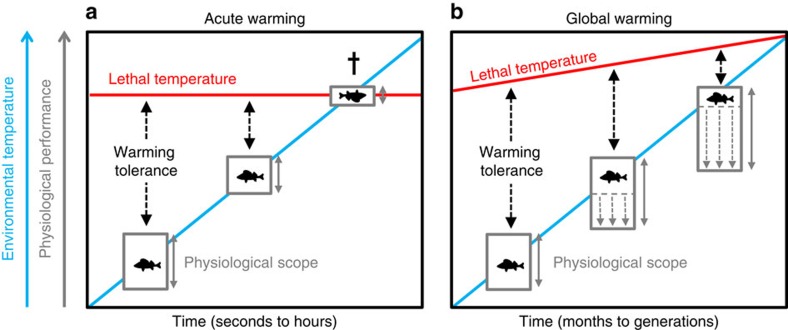
Conceptual model summarizing the hypothesis of ‘Plastic floors and concrete ceilings'. The figure illustrates the differences in thermal effects on eurythermal fishes during acute warming (**a**) and global warming (**b**). The blue line represents the environmental water temperature and the upper red line represents the lethal temperature limit (that is, CT_max_). The difference between these thermal limits is the warming tolerance. Boxes represent basal and maximum physiological functions such as oxygen consumption rate, heart rate and cardiac output at given temperatures, with the difference between lower (basal) and upper (maximum) boundaries representing the scope (grey vertical double arrows). In the acute warming scenario, basal physiological functions increase while the scope may decrease if the increases in basal rates are not matched by similar increases in maximum rates. In the global warming scenario, eurythermal fishes can maintain or even increase physiological scopes by thermally compensating basal rates through thermal plasticity of biochemical and physiological functions, for example, although maximum capacities appear more rigid. The upper lethal temperature limit shows limited thermal plasticity and increases much less than the environmental warming. Thus, even if organismal function and physiological scopes may be adequate with global warming in eurythermal fishes, the warming tolerance is markedly reduced making the animal considerably more vulnerable to episodic heat waves reaching lethal limits. In this model, the ‘plastic floors' are represented by basal physiological rates, whereas the ‘concrete ceilings' are represented by maximum physiological capacities and lethal temperature limits.

The present study shows how a eurythermal temperate fish species has adjusted key physiological traits to successfully maintain its rate of life under chronically heated conditions resembling a severe future climate warming scenario. In addition, our results provide key insights into potential physiological constraints and give rise to a conceptual model outlining how eurythermal fishes deal with changes in environmental temperature. Fish subjected to an acute warming event, such as a transient heat wave, exhibit an elevated basal energy requirement (that is, 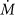
_2rest_) accompanied by a significantly elevated resting heart rate and reduced heart rate scope (Fig. 4a). Although such responses could reduce organismal fitness and competitive performance in the short term, chronic climate warming is considerably slower and should allow for physiological adjustments (for example, acclimation and adaptation) to the warmer environment (Fig. 4b). Indeed, the chronically warmed perch had greatly compensated basal energy requirements and were not aerobically constrained as cardiorespiratory scopes including heart rate scope were restored, even at peak summer temperatures. Nonetheless, our comparison of acutely and chronically heated perch highlights that the thermal plasticity allowing the maintenance of key physiological scopes is primarily achieved through mechanisms that follow principles of ‘plastic floors and concrete ceilings', where the thermal plasticity of resting cardiorespiratory functions is much more pronounced than the plasticity in maximum cardiorespiratory capacities.

Despite the maintained cardiorespiratory performance and improved growth rate of perch in the chronically heated Biotest environment, the acute warming tolerance (that is, CT_max_*−T*_hab_) of these fish progressively diminishes with increasing environmental temperature (Fig. 4b). This finding is of concern because it highlights the reduced thermal buffer of chronically warmed fish for tolerating transient heat waves, a phenomenon expected to become more frequent and severe with ongoing climate change^27^,^28^. Notably, as CT_max_ may be even lower as the rate of warming is decreased further^29^, the estimates of perch warming tolerance presented here may represent a best case scenario.

The concept of ‘plastic floors and concrete ceilings' presented here provides a theoretical framework on how fish adjust physiologically to global warming. Our findings suggest that studies examining physiological ‘floors' in isolation will underestimate the true sensitivity of a population to climate change. We propose that future research attempting to predict the impacts of climate change on fish populations must include measurements of physiological ‘ceilings', including warming tolerance limits over temporal scales that are relevant to the study system.

## Methods

### Study area and collection of experimental animals

The study was conducted at the Biotest enclosure located in the Baltic Sea near the nuclear power plant in Forsmark, Sweden (Fig. 1a). The Biotest enclosure is a man-made ∼1 km^2^ enclosed area in the archipelago that was built in the late 1970s to study the effects of thermal pollution from power plants on aquatic biota^21^. Heated cooling water from two of the nearby nuclear reactors has been continuously pumped into the Biotest enclosure at a rate amounting to ∼90 m^3^ s^−1^ since 1980, maintaining the water temperature around 5–10 °C above that of the water in the surrounding archipelago throughout the year (Supplementary Table 1 and Fig. 1c). The enclosure has a maximum depth of 5 m and an average depth of 2.5 m. Thus, the relatively shallow nature of the enclosure in combination with the high water turnover rates lead to a homogenous thermal environment^30^. Until 2004, a 15-mm grating was positioned at the outflow of the Biotest enclosure, preventing migration of fish larger than ∼10 cm between the inside and outside of the enclosure. The removal of the grating in 2004 theoretically created a less isolated system. Nevertheless, the high water flow at the overflow from the Biotest enclosure to the Baltic Sea has probably always made it very challenging for fish of all sizes and species to migrate in an out of the enclosure^22^.

Adult perch (*Perca fluviatilis, L.*) of mixed sexes were collected using hook and line throughout August and September in 2012 and 2013 (see Supplementary Table 3 for details on experimental fish body characteristics and temperature conditions during individual experimental series). These sampling periods coincided with, or immediately followed, summer peak water temperatures in the area (see Fig. 1c and Supplementary Table 1). Chronically heated fish were collected from the inlet into the Biotest enclosure, whereas reference fish living under natural thermal conditions were caught at the water intake channel to the nuclear power plant where the temperature is the same as the surrounding archipelago (see Fig. 1a for details). Following capture, fish were transported (<3 km) to a wet lab facility beside the Biotest enclosure and held until experimentation in 1,200 l tanks supplied with flow-through aerated Baltic Sea water (salinity ∼5 p.p.m.) at their respective environmental temperature (Supplementary Table 3). Fish were not fed in captivity and at least 3 days of recovery were allowed following capture before the experiments commenced. In order to minimize the risk for unforeseen time effects during the experiments, individuals from the different experimental groups were randomly selected during the course of the study. Animal care and all experimental procedures were performed in accordance with the guidelines and regulations approved by the Ethical Committee of Gothenburg Sweden (ethical permit 65-2012).

### Experimental protocols

In all experiments, water temperatures were recorded continuously using custom-built digital thermometers with analogue outputs (EW 7221, Crn Tecnopart) calibrated against a precision thermometer (testo 735, Nordtech Instruments AB). Experimental data were sampled using Power Lab units (ADInstruments Pty Ltd) connected to portable computers running LabChart Pro software (ADInstruments Pty Ltd) unless otherwise stated. Details on experimental temperature conditions in individual experimental series are presented in Supplementary Table 3.

### Oxygen consumption rate and aerobic scope experiment

Oxygen consumption rates (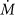
_2_) of perch were measured using best practices in intermittent flow-through respirometry according to Clark *et al*.^7^ in order to assess metabolic thermal plasticity. Resting oxygen consumption rate (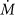
_2rest_) and maximum oxygen consumption rate (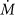
_2max_) were measured in three individual groups of uninstrumented fish as outlined below. Reference fish were tested at reference temperature (18 °C), as well as 24 h after being acutely warmed to the temperature of the Biotest enclosure (23 °C; that is, ‘acutely warmed fish'), whereas fish from the Biotest enclosure were only tested at their respective environmental temperature (23 °C; that is, ‘chronically warmed fish', see Supplementary Table 3). Fish were introduced into one of eight size-matched intermittent flow-through horizontal cylindrical perspex respirometers at either reference or Biotest temperature. Fish were maintained at the test temperature overnight (>10 h), while 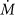
_2_ was measured every 15 min between automated flush cycles (see Fig. 3b of ref. 7). The oxygen concentration of the water in the respirometer was measured continuously at 0.5 Hz using a FireSting O_2_-optode system (PyroScience), and 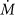
_2_ was calculated from the decline in oxygen concentration in the respirometers between flush cycles using equation 2 in Clark *et al*.^7^. 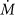
_2rest_ was calculated as the mean of the lowest 10% of 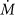
_2_ values measured during this >10 h period. The following day, each fish was individually removed from the respirometer to undergo an exhaustive exercise protocol consisting of a 3-min period of manual chasing performed in a circular tank (diameter 1.2 m, water depth 20 cm) containing aerated water at the experimental temperature. 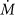
_2_ measurements commenced within 20 s of the fish concluding the exercise protocol and continued for 1–2 h post exercise. The highest 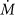
_2_ measured during this period (which typically occurred during the first minutes after re-entry into the respirometer) was designated 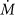
_2max_. AS was subsequently calculated as 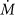
_*2max*_–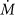
_*2rest*_. This exercise protocol was selected after several pilot experiments revealed that the perch would not swim steadily or consistently in a swim tunnel respirometer. Any form of maximal exercise (whether initiated via a chase protocol or a maximal swim tunnel test) involves a certain degree of anaerobic metabolism that is repaid after the exercise (that is, excess post-exercise oxygen consumption), and it is clear for fishes of a similar ecotype as perch that the highest measurements of 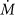
_2_ are typically obtained immediately after an exhaustive exercise protocol rather than during the exercise itself^31^.

### Cardiovascular scope experiment

To assess the thermal plasticity of cardiovascular function, the same experimental groups as those used to assess oxygen consumption rate and AS were used, but in different surgically instrumented fish (see Supplementary Table 3 for details). Fish were anaesthetized in Baltic Sea water containing MS-222 (100 mg l^−1^; Sigma) and placed on a surgery table covered with damp foam. The gills were continuously irrigated with re-circulating aerated seawater (∼8 °C) containing a lower dose of MS-222 (50 mg l^−1^) to maintain anaesthesia. The ventral aorta was cannulated with a PE-31 or PE-50 catheter filled with heparinized (20 IU ml^−1^) 0.9% saline that was occlusively implanted into the third afferent branchial artery and advanced into the vessel until the tip was located close to the bifurcation of the ventral aorta according to Axelsson and Fritsche^32^. Correct placement was verified on the surgery table by positive pressure and unhindered withdrawal of blood. Ventral aortic blood pressure was measured by connecting the saline-filled catheter to a DPT-6100 pressure transducer (pvb Medizintechnik) calibrated against a static water column and the water surface in the experimental tank serving as a zero pressure reference. The signal from the transducer was amplified using a 4ChAmp pre-amplifier (Somedic). To measure cardiac output, the ventral aorta was dissected free anterior to the heart, taking care not to damage the pericardium or nearby nerves and vessels. A Transonic transit-time blood flow probe (sizes: 1.5–2.5 mm; Transonic Systems Inc) was fitted around the vessel^33^. The Transonic blood flow probes were connected to a Transonic blood flow-meter (model T206; Transonic System Inc. ). Transonic probes were individually bench-calibrated according to the manufacturer's instructions to account for potential zero offsets and to compensate for temperature effects on probe readings. The catheter and flow probe leads were attached with silk sutures to the skin. Fish were then placed individually in appropriately sized holding tubes that were placed in a larger outer tank, supplied with a continuous flow of aerated seawater and covered with black plastic to minimize visual stimuli. Fish were given at least 24 h of post-surgical recovery.

At the beginning of each experiment, baseline cardiovascular variables were recorded continuously for at least 2–3 h and values were taken during periods when the heart rate was low and stable. Each fish was then removed from the holding tube and subjected to a similar manual chase protocol as the fish involved in the oxygen consumption measurements outlined above, the only difference being that the trailing catheters and leads necessitated the use of a smaller size chasing tank (400 × 600 mm^2^). Subsequently, each fish was immediately returned to its holding tube to record the maximum cardiovascular response (based on cardiac output), which occurred immediately after the enforced exercise protocol.

The blood flow data were normalized to body mass to obtain mass-specific values for cardiac output. Heart rate was obtained from pulsatile blood pressure or flow records and the cyclic blood pressure signal was analysed off-line to determine the mean blood pressure using the blood pressure module in the LabChart Pro software (ADInstruments Pty Ltd). Cardiac stroke volume was calculated as cardiac output divided by heart rate. The total vascular resistance was calculated as blood pressure divided by cardiac output, assuming that central venous blood pressure is zero in fish^34^. Cardiac power output (mW g^−1^ ventricular mass) was calculated as the product of ventral aortic blood pressure and cardiac output divided by the ventricular mass^35^.

### Cardiac autonomic control experiment

Individual groups of fish from both populations were anaesthetized as described above and equipped with a ventral aortic catheter only. They were held in experimental tubes supplied with flow-through aerated seawater at their respective environmental temperatures (Supplementary Table 3) and were given a post-surgical recovery period of at least 24 h. Baseline blood pressure and heart rate were recorded for at least 2–3 h. Cardiac autonomic tones were then determined using the protocol of Altimiras *et al*.^36^. First, atropine sulphate (1.2 mg kg^−1^; Sigma) was injected to block muscarinic receptors and cardiovascular variables were allowed to stabilize for at least 30 min. Subsequently, sotalol hydrochloride (2.7 mg kg^−1^; Sigma) was injected to block β-adrenergic receptors and cardiovascular variables were allowed to stabilize for at least another 20 min before recordings were taken.

The heart rate after complete autonomic blockade is the intrinsic heart rate, which reflects the spontaneous activity of the cardiac pacemaker irrespective of extrinsic autonomic input. When the intrinsic heart rate had been determined at the respective environmental temperatures, the temperature was changed over ∼1 h to the ‘opposite' temperature, which meant that reference fish were exposed to Biotest temperature and fish from the Biotest enclosure were exposed to reference temperature to determine the acute effects of temperature on the intrinsic heart rate (see Supplementary Table 3 for details on experimental temperatures). Atropine and sotalol treatment lead to expected increases and decreases in heart rate, respectively, in both experimental groups. Yet, the heart rate responses to both drugs were more pronounced in the chronically warmed Biotest fish (Supplementary Fig. 2). Percentage cardiac cholinergic and adrenergic tones at the respective environmental temperatures for both populations of fish were calculated from changes in the heart beat interval (*interval=(60/heart rate*)) as described by Altimiras *et al*.^36^. Briefly, cholinergic tone was calculated as ‘(*untreated interval–atropine treated interval)/intrinsic interval*' and adrenergic tone was calculated as ‘(*intrinsic interval–atropine treated interval)/intrinsic interval*'. The intrinsic interval is the heart beat interval after complete autonomic blockade.

### Thermal tolerance experiment

To examine how whole animal acute thermal tolerance and cardiovascular functions are affected under chronically heated conditions, reference and Biotest fish were anaesthetized as described above and instrumented with tight-fitting custom made Perspex cuff-type Doppler flow probes (1.6–2.0 mm; Iowa Doppler products) around the ventral aorta^37^. Fish were then held individually in opaque experimental boxes receiving aerated seawater, maintained at 19 °C using a custom-built electronic thermostat and a 2-kW heating element in a separate header tank, and given at least 24 h of post-surgical recovery. The experimental setup was shielded by black plastic to avoid disturbance of the fish, and the fish were monitored using a camera mounted above the experimental setup. Doppler flow probes were connected to a directional-pulsed Doppler flow meter (model 545C-4, University of Iowa). Doppler flow probes were chosen in these experiments as they are considerably smaller than the Transonic probes and have thinner and more flexible leads, which were anticipated to interfere minimally with the fish during the temperature protocol. However, the Doppler technique does not give absolute values for blood flow and so cardiac output data from the Doppler flow meter were expressed as relative changes from 19 °C, with the starting value in reference fish set to 100% and the starting value for Biotest fish estimated to be 88% at this temperature. This estimate was calculated from absolute values of cardiac output obtained using Transonic blood flow probes as outlined above in reference fish at 17 °C (Supplementary Table 1) and preliminary data for a group of Biotest fish examined at the same temperature (20.5±2.1 ml min^−1^ kg^−1^, *n*=12). Thus, inherent to this estimate was the assumption that the difference between experimental groups was the same at 17 and 19 °C.

Cardiac output was recorded continuously for at least 2–3 h at 19 °C to ensure stable baseline values. The temperature was then increased in steps of 1 °C per 20 min (that is, a heating rate of 3 °C h^−1^) with continuous measurements of cardiac output. This heating regime was chosen as preliminary experiments with a thermocouple implanted into the deep dorsal muscles of perch revealed that this temporal protocol was sufficient for fish of this size to reach thermal equilibrium with the surrounding water and for cardiac variables to stabilize, while minimizing the potential effects of thermal acclimation to the higher temperatures^29^. Oxygen levels in the experimental setup were always close to full saturation. The temperature was increased until the righting reflex was lost for 3 s, which was considered to represent the critical thermal maximum (CT_max_) according to the definitions by Lutterschmidt and Hutchison^38^. As expected, blood flow increased continuously during the acute temperature increase, mainly due to an increase in heart rate (Supplementary Fig. 3). At CT_max_, the fish were quickly removed from the experimental setup and killed by a sharp blow to the head. We estimated the warming tolerance in individual fish as *CT*_*max*_*−maximal habitat temperature*^15^. In this case, the maximal habitat temperature was estimated by calculating the mean of daily mean temperatures during the five warmest days over 3 weeks before the determination of CT_max_ using raw data from Fig. 1c.

### Determination of growth rate and post mortem tissue analysis

In 2012, otoliths and opercular bones were collected following termination of cardiovascular experiments. The left operculum and otolith were removed, cleaned in freshwater and left to dry in air. To mark the annual rings, the otoliths were heated over an open flame until obtaining a light brown colour. They were thereafter broken right through the centre and placed in water. To determine the age of the fish, the surface of the fracture was examined under a stereo microscope (magnification: × 20–50) and the annual rings were counted from the centre to the edge of the otolith. To determine yearly growth, the opercular bone was placed in propylene glycol to enhance the annual rings. To determine the growth rate, the distance between the annual rings was measured using a digital caliper and yearly size was obtained by back calculating according to standard procedures at the Swedish Institute of Coastal Research.

To examine potential differences in gross organ mass between populations, relative organ masses were determined for most of the fish in 2012. The heart ventricle, the spleen and the liver were removed. Fat and connective tissues were carefully trimmed off and the organ was thoroughly blotted dry on tissue paper before the wet mass was determined. Body condition was calculated according to the formula condition=100*weight/(fork length^3^), where weight is in grams and length is in centimetres.

### Data analyses and statistics

The overall objective of our statistical analysis was to compare the effects of acute (reference fish heated to Biotest temperature for 24 h) and chronic (fish from Biotest enclosure) environmental temperature increases on cardiorespiratory variables relative to the reference fish at natural temperature conditions, and to identify physiological and morphological differences between the two populations.

Significant differences among groups were identified using either a one-way analysis of variance for the cardiovascular variables or an analysis of covariance for the respiratory variables with mass as a covariate (see Fig. 2a–c and Supplementary Table 4). A square root transformation was applied to stroke volume and total vascular resistance before analysis as these variables did not meet the assumptions for a normal distribution according to the Shapiro–Wilks test or constant variance according to the Levene's test. We also assessed the degree of thermal compensation of rate-dependent cardiorespiratory functions including 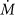
_2rest_ and cardiac output (that is, the difference in thermal response between acutely heated reference fish and chronically heated Biotest fish relative to reference fish), by analysing *Q*_10_ relationships for the thermal responses using the van't Hoff equation^3^.

Direct comparisons between the two populations were performed using a two-tailed Student's *t*-test for intrinsic heart rate (Fig. 2d), autonomic tones (Fig. 2e), CT_max_ (Fig. 3), morphological characteristics and age (Supplementary Table 2). For dependent data, including yearly growth (Supplementary Fig. 1) and cardiovascular variables during the thermal tolerance test (Supplementary Fig. 3), a linear mixed model was used. In the linear mixed models, individual fish were set as the subjects, whereas the repeated measures were age and year of life in the growth analysis (Supplementary Fig. 1) and temperature in the thermal tolerance test (Supplementary Fig. 3). AR(1) was used as the type of repeated covariance because recordings that were close in time also were more dependent than more temporally distant recordings. Fish origin (that is, reference or Biotest fish) was included in the model as a possible explanatory factor. *P*-values were adjusted for multiple testing using the Holm–Bonferroni method. All data are presented as means (±s.e.) unless otherwise stated. Statistical analyses were conducted in SPSS 20 for Windows (SPSS Inc.). Differences where *P*<0.05 were regarded as statistically significant.

## Additional information

**How to cite this article**: Sandblom, E. *et al*. Physiological constraints to climate warming in fish follow principles of plastic floors and concrete ceilings. *Nat. Commun.* 7:11447 doi: 10.1038/ncomms11447 (2016).

## Supplementary Material

Supplementary Information Supplementary Figures 1-3 and Supplementary Tables 1-4.
