# Supplementary material for: Physiological constraints to climate warming in fish follow principles of plastic floors and concrete ceilings
Source: Nat Commun. 2016 May 17;7:11447. doi: 10.1038/ncomms11447 (PMC4873662; doi:10.1038/ncomms11447)
Supplement: Supplementary Information — Supplementary Figures 1-3 and Supplementary Tables 1-4. [file ncomms11447-s1.pdf]

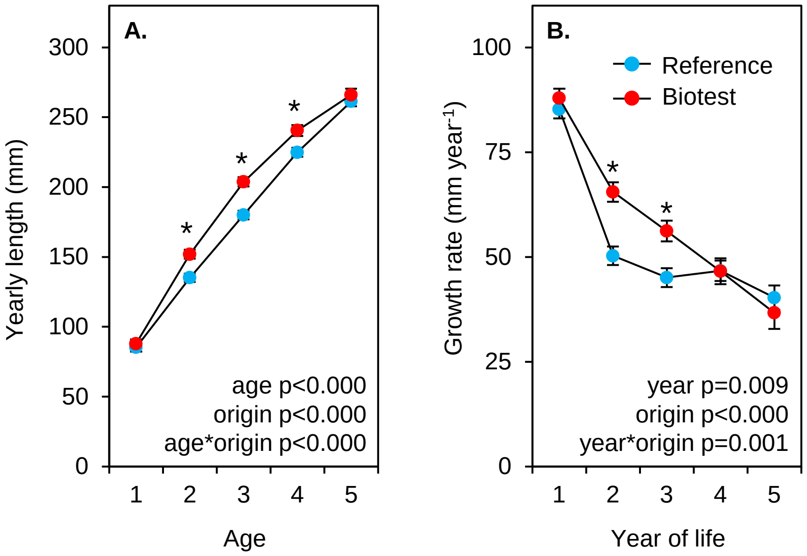

Supplementary Figure 1. Yearly length and growth rate. Variables are length (A) and growth rate (B) in reference (n=30-52, blue) and chronically warmed Biotest (n=17-49, red) perch (*Perca fluviatilis, L.*) sampled in August and September 2012. Values are means (±SE). Included in the figures are the fixed effects from the mixed model analysis and * represents significant differences between groups at specific time points (p≤0.05).

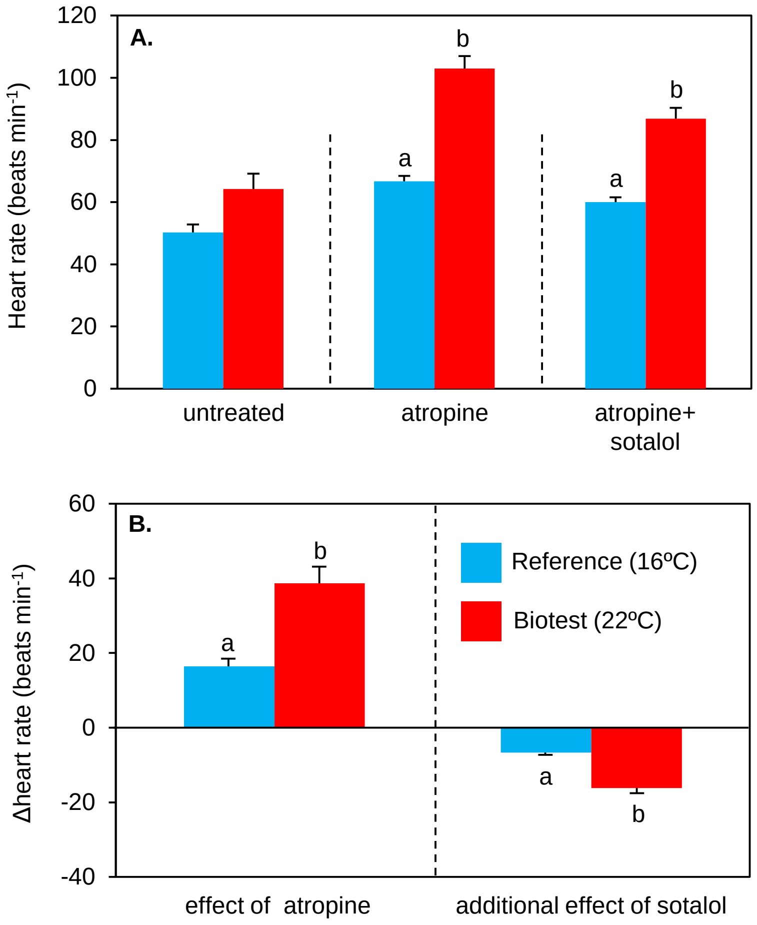

Supplementary Figure 2. Cardiovascular responses to pharmacological blockade. Panel A shows resting heart rate in reference (blue, n=13) and chronically warmed Biotest (red, n=9) perch (*Perca fluviatilis*) under untreated conditions, following muscarinic receptor blockade with atropine (1.2 mg kg^{-1}), and following additional β-adrenoceptor blockade with sotalol (2.7 mg kg^{-1}). Variables were recorded at the respective environmental temperatures and the heart rate after double autonomic blockade (atropine+sotalol) is the intrinsic heart rate. Panel B shows the changes in heart rate induced by the respective pharmacological treatments. Values are means (±SE). Dissimilar letters denote significant differences between groups ($p \leq 0.05$; two-tailed student t-test).

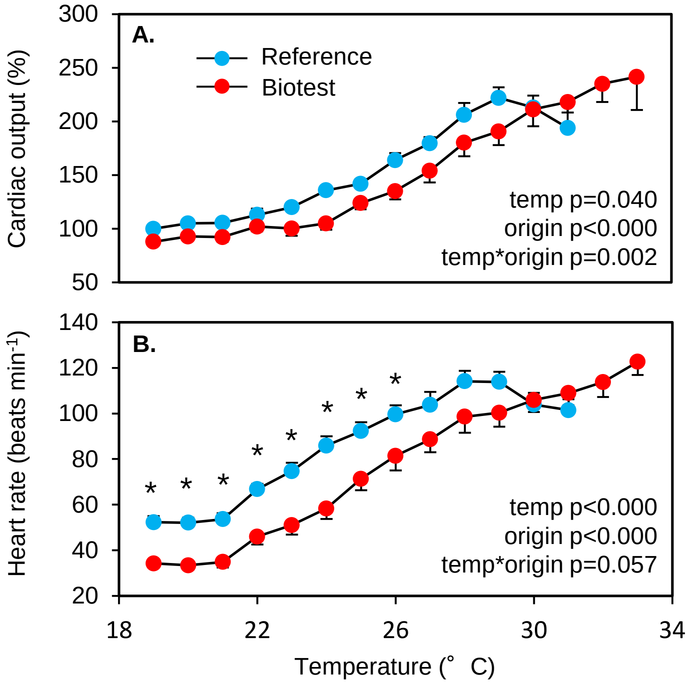

Supplementary Fig. 3. Cardiac responses to acute warming. Variables are cardiac output (A) and heart rate (B) during CT_{max} determination (heating rate: 3°C h^{-1}) in reference (blue, n=11) and chronically warmed Biotest (red, n=11) perch (*Perca fluviatilis, L.*). Only recordings up to 31°C were used in the analysis as sample size decreased as individuals reached CT_{max}. Values are means (±SE). Included in the figures are the fixed effects from the mixed model analysis and * represents significant differences between groups at specific temperatures (p≤0.05).

Supplementary Table 1. Summary of water temperatures in the Forsmark area in 2012 and 2013.

	Environmental temperatures (°C)		
	Experimental period mean	*Annual mean*	*Annual range*
2012 (7 Aug – 30 Aug)			
Reference	16.0±1.4	7.9±6.3	-0.2 – 20.7
Biotest	22.0±1.9	15.6±5.8	7.7 – 28.5
ΔT	6.0±1.0	7.7±1.2	-
2013 (25 Aug – 11 Sept)			
Reference	17.6±0.4	8.0±6.5	-0.2 – 18.5
Biotest	23.1±2.0	16.0±5.8	8.1 – 26.3
ΔT	5.5±2.2	8.0±1.5	-

Environmental temperature values are based on daily mean temperature records at 2 m depth in the cooling water intake channel (*Reference*) and at the centre of the Biotest enclosure (*Biotest*). Values are expressed as means (±SD). *ΔT* represents the temperature difference between locations and annual temperature ranges are expressed as the coldest and warmest days, respectively. The experimental periods represent the periods when experiments were performed.

Supplementary Table 2. Morphological characteristics and age in perch (*Perca fluviatilis, L.*).		
	Reference	*Biotest*
n	45-55	45-50
Body mass (g)	363.6±17.6	327.1±15.7
Body mass range (g)	145 - 780	165 - 638
Fork length (mm)	301.7±4.5	293.1±4.0
Fork length range (mm)	230 - 389	240 - 368
Condition index	1.27±0.01	1.25±0.02
Age (years)	5.3±0.3	4.2±0.2*
Relative ventricle mass (%)	0.06±0.00	0.05±0.00*
Relative spleen mass (%)	0.17±0.01	0.13±0.01*
Relative liver mass (%)	1.21±0.03	1.00±0.02*
Fish were collected from the reference area (*Reference*) and the biotest enclosure (*Biotest*) throughout August and September 2012. Data are pooled from fish used in cardiorespiratory experiments. Values are means (±SE). Asterisks denote significant difference between experimental groups ($p \leq 0.05$; two-tailed student t-test).		

Supplementary Table 3. Summary of body characteristics for perch (*Perca fluviatilis, L.*) and temperature conditions during individual experimental series in 2012 and 2013.

Experimental series	*n*	*Body mass (g)*	*Fork length (mm)*	*Test temperature (°C)*	*Holding temperature (°C)*
Oxygen consumption rate and aerobic scope (2013)					
Reference	9	258.1±43.3	256±15	18.2±0.0	18.7±1.1
Reference acutely warmed (24 h)	10	312.0±77.4	262±14	23.3±0.2	18.7±1.1
Chronically warmed (Biotest)	10	284.9±39.0	265±11	23.3±0.2	23.7±0.9
Cardiovascular scope (2012)					
Reference	7	289±23	285±9	17.4±0.8	16.5±1.3
Reference acutely warmed (24 h)	10	277±19	280±7	21.8±1.1	16.5±1.3
Chronically warmed (Biotest)	10	269±17	278±6	22.2±1.6	21.7±1.5
Cardiac autonomic control (2012)[1]					
Reference	13	417±48	313±12	15.5±0.7	15.5±1.1
				(22.4±0.5)	
Chronically warmed (Biotest)	9	377±42	304±10	22.2±0.4	21.0±2.1
				(15.7±0.4)	
Thermal tolerance (2012)[2]					
Reference	16 (11-4)	400±23	312±6	-	17.3±1.0
Chronically warmed (Biotest)	16 (11-4)	388±31	307±8	-	22.1±1.7

[1] Temperatures within parentheses represent the temperatures after acute temperature change where intrinsic heart rate was determined. [2] n-values within parentheses represent number of animals where reliable cardiovascular recordings were obtained, presented in reverse numerical order because n decreased as individuals reached their CT_{max}. Morphological characteristics are expressed as means (±SE) and temperatures are expressed as means (±SD).

Supplementary Table 4. Cardiorespiratory variables in perch (*Perca fluviatilis, L.*) from two populations (Reference and Biotest) measured at reference temperatures (17-18°C) or Biotest temperatures (22-23°C).			
	Reference	*Reference acutely warmed (24 h)*	*Chronically warmed (Biotest)*
Test temperature (°C)	17-18	22-23	22-23
Oxygen consumption rate (mg O_2 h^{-1} kg^{-1})			
n	9	10	10
Resting	91.3±5.9[a]	135.2±6.2[b]	111.2±3.5[c]
Maximum	303.6±22.0[a]	353.7±17.5[b]	359.9±24.5[b]
Scope	212.3±20.4	218.5±12.9	248.7±23.0
Cardiac output (ml min^{-1} kg^{-1})			
n	7	7-10	8-10
Resting	23.2±2.0[a]	31.2±1.8[b]	26.4±1.3[ab]
Maximum	33.8±3.3[a]	52.2±2.3[b]	51.0±4.7[b]
Scope	10.7±3.9	20.6±2.5	23.3±3.8
Heart rate (beats min^{-1})			
n	7	7-10	8-10
Resting	44.4±4.5[a]	89.2±3.3[b]	60.9±3.7[c]
Maximum	62.6±3.7[a]	81.4±4.3[b]	86.6±4.8[b]
Scope	18.2±3.5[a]	-7.5±6.9[b]	27.4±3.3[a]
Stroke volume (ml)			
n	7	7-10	8-10
Resting	0.55±0.07[a]	0.35±0.02[b]	0.45±0.04[ab]
Maximum	0.56±0.07	0.65±0.05	0.60±0.06
Scope	0.01±0.07[a]	0.30±0.05[b]	0.12±0.05[ab]
Ventral aortic blood pressure (cm H_2O)			
n	7	7-10	8-10
Resting	53.0±1.2	53.5±0.9	53.0±1.4
Maximum	63.0±1.5	62.5±1.8	64.4±2.4
Scope	10.0±1.8	9.2±2.5	11.6±1.6
Total vascular resistance (cm H_2O ml^{-1} min^{-1} kg^{-1})			
n	7	7-10	8-10
Resting	2.4±0.2[a]	1.8±0.1[b]	2.1±0.1[ab]
Maximum	2.0±0.2[a]	1.2±0.5[b]	1.3±0.1[b]
Scope	-0.4±0.2	-0.5±0.1	-0.6±0.1
Cardiac power output (mW g $ventricle^{-1}$)			
n	7	7-10	8-10
Resting	3.3±0.3	4.5±0.3	4.4±0.4
Maximum	5.7±0.5[a]	9.1±0.9[b]	10.5±1.1[b]
Scope	2.4±0.7[a]	4.5±0.8[ab]	5.7±0.8[b]
Variables are presented for quiescent fish under resting conditions (*Resting*) and when oxygen consumption rate was maximal (*Maximum*). *Scope* is the difference between resting and maximum values. '*Reference*' and '*Chronically warmed (Biotest)*' fish were measured at their respective environmental temperatures and '*Reference acutely warmed (24 h)*' are reference fish tested after acute exposure to Biotest temperature. Respiration variables were measured in uninstrumented fish. All variables are expressed as means (±SE). Dissimilar letters denote significant differences between experimental groups (p≤0.05; one-way ANOVA for all cardiovascular variables and ANCOVA with body mass as covariate for oxygen consumption rate).			
